# Comparison and Optimization of Generalized Stamping Machine Fault Diagnosis Models Using Various Transfer Learning Methodologies

**DOI:** 10.3390/s25061779

**Published:** 2025-03-13

**Authors:** Po-Wen Hwang, Yuan-Jen Chang, Hsieh-Chih Tsai, Yu-Ta Tu, Hung-Pin Yang

**Affiliations:** 1 Department of Aerospace and Systems Engineering, Feng Chia University, Taichung City 407102, Taiwan; pwhwang@fcu.edu.tw; 2 Master’s Program of Data Science, Feng Chia University, Taichung City 407102, Taiwan; tsaishihchih@gmail.com; 3 Chin Fong Machine Industrial Co., Ltd., Changhua City 500031, Taiwan; yuta2@chinfong.com.tw (Y.-T.T.); besn@chinfong.com.tw (H.-P.Y.)

**Keywords:** stamping production, data analysis, predictive maintenance, deep learning, generalized model

## Abstract

The integration of artificial intelligence (AI) with stamping technology has become increasingly critical in smart manufacturing, driven by advancements in both fields. Total clearance, a crucial determinant of both process and product quality in stamping operations, significantly impacts cutting precision, material deformation, and the longevity of stamping equipment. Consequently, real-time monitoring and prediction of total clearance are essential for effective process control and fault diagnosis. However, the heterogeneity of stamping machine designs necessitates the development of numerous machine-specific models, posing a significant challenge for practical implementation. This research addresses this challenge by developing a generalized fault diagnosis model applicable across multiple stamping machine types. Specifically, the model is designed to monitor four distinct machine models: OCP-110, G2-110, G2-160, and ST1-110. Vibration data, acquired using accelerometers strategically placed at two distinct sensor locations on each machine, serve as the primary input for the model. Four prominent deep learning architectures—a 10-layer convolutional neural network (CNN), a CNN with residual connections (CNN-Res), VGG16, and ResNet50—were rigorously evaluated in conjunction with fine-tuning strategies to determine the optimal model architecture. The resulting generalized fault diagnosis model achieved an average accuracy, recall rate, and F1 score exceeding 99%, demonstrating its efficacy and reliability for real-world applications. This proposed approach offers the potential for scalability to additional stamping machine types and operational conditions, thereby streamlining the deployment of predictive maintenance systems by equipment manufacturers.

## 1. Introduction

Servo presses are widely employed in manufacturing, particularly in the hardware, automotive, and 3C industries, due to their stable forming quality and high operational efficiency, especially for metal forming. However, prolonged high-load operation, coupled with factors such as high-frequency operation, vibration, and thermal expansion, can gradually alter press clearances. These alterations directly impact stamping accuracy, product quality, and equipment lifespan, ultimately reducing production line stability and efficiency.

In line with Industry 4.0 objectives, manufacturers are increasingly pursuing automated production lines, including fully automated, unmanned factories. In such environments, continuous operation and high product quality are paramount. Consequently, real-time monitoring of press health, fault diagnosis, and immediate alerts during stamping are essential.

Ge et al. employed a support vector machine (SVM) with three different kernel functions for fault diagnosis in sheet metal stamping, achieving over 93% accuracy in both blanking and progressive stamping operations [1]. This surpassed the 90% accuracy achieved using an artificial neural network (ANN), while requiring fewer training samples.

Bassiuny et al. proposed a fault classification method combining empirical mode decomposition (EMD) and a learning vector quantization (LVQ) network [2]. Strain signals were decomposed into intrinsic mode functions (IMFs) using EMD, and the Hilbert marginal spectrum, reflecting operating conditions and fault patterns, was used as input for the LVQ classifier. This method achieved a 96.3% success rate in detecting artificially induced defects across 107 experiments.

Özcan and Fığlalı et al. demonstrated the superiority of artificial neural networks (ANNs) over multi-linear regression and other cost estimation models for predicting stamping die costs [3].

Chen et al. proposed a deep neural network (DNN)-based method for estimating the remaining useful life (RUL) of stamping presses by analyzing punch sounds [4]. Their findings highlighted the effectiveness of working sounds as features for machinery health monitoring and demonstrated the potential of employing sampling rate reduction and a novel NA-MFCC feature extraction technique to reduce computational complexity.

Chang et al. investigated the impact of three common stamping press faults (lack of lubrication oil, oil quality variation, and clearance variation) on workpiece quality and die/press lifespan [5]. Data acquisition involved capturing three-phase currents and tri-axial vibration signals using an NI DAQ system. From the 72 generated features, logistic regression and principal component analysis (PCA) were employed to select the top three key features for a health assessment model. This model triggers early warnings based on a predefined health indicator threshold. Fault diagnosis, achieving up to 99% accuracy, was performed using SVM, K-NN, and XGBoost classification algorithms.

Yuan et al. proposed a novel fault diagnosis method for forging presses, termed the multi-modal attention-based convolutional neural network (CNN) with PCA and a transductive support vector machine (TSVM) (MACNN-PTSVM), which integrated the CNN, PCA, and transductive support vector machine (TSVM) [6]. This approach effectively integrates multimodal information from 12 sensor signals, including displacement, cylinder pressure balance, impact force, and hydraulic brake pressure signals. The MACNN-PTSVM model, which incorporates an attention mechanism, CNN, PCA, and TSVM, processes smoothed monitoring signal data to extract fault features. The model offers substantial advantages in classification performance. Notably, its semi-supervised learning capability exhibits superior generalization and diagnostic accuracy, particularly when handling unlabeled data.

As highlighted by Yuan et al. [6], achieving model generalization is crucial for real-world deployment in production lines. Stamping lines often comprise diverse press models with varying structures, configurations, and operating conditions. Constructing individual models for each machine type and operating condition is impractical and cost-prohibitive. Similarly, press manufacturers face challenges in deploying a universal model across diverse product ranges, which include different models, tonnage capacities, and operating conditions. Although dedicated models for each machine may improve accuracy, their proliferation complicates management, increases operational complexity, and diminishes cost-effectiveness. Furthermore, even with dedicated models, client-specific customization of operating conditions renders pre-built models ineffective and necessitates onsite fine-tuning.

Building on this background, we propose to explore the application of transfer learning and multi-condition model flattening techniques. Transfer learning facilitates the use of the knowledge of existing conditions to improve model adaptability to new scenarios. Simultaneously, multi-condition model flattening allows us to fine-tune a base model to enhance its accuracy and robustness across diverse operating conditions. This approach is promising for improving both the performance and practicality of fault diagnosis systems in stamping presses.

## 2. Background and Preliminaries

### 2.1. Problem Statement

Sheet metal stamping processes are widely used in various industries owing to their high precision and efficient production rates. However, it is considerably challenging to monitor these processes because the acquired signals are often non-stationary transients. Analyzing vibration signals from different stamping machines further complicates the task because their characteristics can vary substantially.

**Figure 1:**
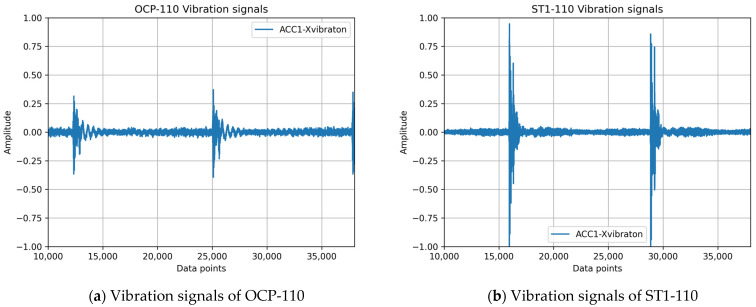

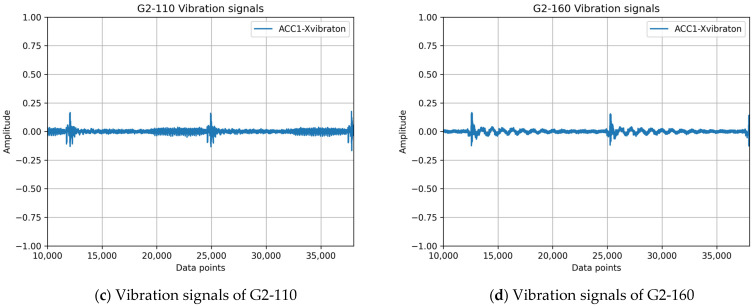
Vibration signals of four different stamping press models under identical operating conditions.

Figure 1 illustrates the vibration signals of four different stamping machine models during individual stamping operations: (a) C-frame single-crank power press OCP-110, (b) straight-side single-crank precision press ST1-110, (c) C-frame double-crank power press G2-110, and (d) G2-160. Even under identical operating conditions, the vibration patterns exhibit substantial variability across these models, which makes it challenging to develop a universal fault diagnosis model that can be applied across diverse stamping machines.

### 2.2. Accelerometer Placement Optimization

Although Yuan et al. [5] suggested utilizing existing sensor signals for fault classification to minimize costs, the sampling rates of these signals in stamping presses are often limited to 10 Hz or less by PLC acquisition systems. Given that the stamping process occurs within approximately 0.2 s, with only about 1.5 s between cycles, such low sampling rates hinder effective fault mode differentiation, especially across diverse press models. This limitation is evident from a domain knowledge perspective.

To enhance fault diagnosis, we utilize low-cost microelectromechanical system (MEMS) accelerometers with high sampling rates to capture detailed vibration data, facilitating improved feature extraction and differentiation of fault modes. Accelerometer placement was optimized through consultation with experienced press design engineers, leading to installation at two critical locations within the press structure to determine the optimal location for developing a generalized fault diagnosis model.

### 2.3. Related Works

Based on the preceding discussion, several key challenges persist in the practical application of fault diagnosis for stamping presses. These include the effective acquisition of press signals using low-cost, minimal-sensor configurations, coupled with machine learning algorithms, to perform fault diagnosis across multiple operational conditions. Crucially, this diagnostic capability should generalize to presses of different models and under varying stamping operational conditions. Addressing these challenges constitutes a primary objective of this research.

Furthermore, in practical press operations, the most prevalent fault is a change in stamping clearance [5]. This deviation leads to anomalies in product quality, resulting in excessive scrap and increased production costs. Therefore, developing a fault diagnosis model capable of detecting press malfunctions caused by clearance variations during long-term stamping operations is essential for ensuring product quality. Notably, the investigation of such a clearance-specific fault diagnosis model has not been addressed in previous studies. Consequently, establishing a clearance fault diagnosis model is another principal objective of this research.

Finally, due to the substantial size of stamping presses, determining the optimal sensor placement for achieving the best diagnostic performance is crucial. While prior research [6] has utilized signals from multiple sensors to construct fault diagnosis models, the question of optimal placement for a single vibration sensor remains unaddressed. Identifying this optimal single-sensor location is the final primary research objective of this study.

To achieve the aforementioned objective of a generalized fault diagnosis model, we draw upon prior research in predictive maintenance [7]. This research demonstrates that employing convolutional neural network (CNN) models within a deep learning classification framework, in conjunction with transfer learning, can yield effective fault diagnosis and classification. This transfer learning approach offers a potential solution to overcome the limitations associated with traditional machine learning and deep learning in predictive maintenance, particularly concerning model generalization across multiple operational conditions.

Furthermore, as highlighted by Chen et al. [8], the development of fault diagnosis models with long-term applicability is of paramount importance due to inevitable factors such as variations in working environments and operating conditions, component performance degradation, and heterogeneity among automated systems. Consequently, transfer learning has emerged as an indispensable tool, endowing fault diagnosis methods with self-learning and adaptive capabilities.

Yao et al. [9] explicitly highlighted the challenge of simultaneously balancing accuracy and efficiency in fault diagnosis models. More complex models typically achieve higher accuracy, while simpler models offer greater efficiency. However, in industrial applications, efficiency is as crucial as accuracy. Consequently, their research focused on developing a fault diagnosis model for rolling bearings operating under varying conditions, proposing a lightweight deep transfer learning (DFL) model termed the parallel multiscale convolutional transfer neural network (PMCTNN). PMCTNN enhances accuracy by optimizing its architecture rather than increasing its depth, thereby maintaining efficiency. This approach allows the resulting fault diagnosis model to exhibit both superior diagnostic accuracy and efficiency.

Furthermore, the substantial time investment required for hyperparameter tuning in transfer learning, to achieve accurate fault diagnosis, presents a practical challenge. Addressing this, Sun et al. [10] introduced a novel approach: cross-domain transfer learning with a fine-tuning mechanism (CTL-FTM). This method is designed for gearbox fault diagnosis using imbalanced datasets, employing a pre-trained model for feature extraction and a shallow network for fault type classification. This demonstrates that achieving high diagnostic accuracy does not necessarily necessitate deeply layered convolutional neural networks; an appropriately designed fine-tuning mechanism can also yield excellent model performance.

Furthermore, traditional deep learning-based fault diagnosis methods typically assume that training and testing data follow identical distributions. This assumption often holds during model training, resulting in a seemingly satisfactory performance. However, upon deployment in real-world production line scenarios, the diagnostic performance frequently falls short of expectations. This discrepancy arises because the data distribution in the actual production line often deviates from that of the training data, leading to a significant degradation in fault diagnosis performance.

To mitigate this issue, Chen et al. [11] introduced the concept of transfer learning into deep learning, leveraging knowledge acquired from other datasets or models. This provides valuable guidance for our objective of applying the developed fault diagnosis model to stamping presses operating under diverse conditions and across different models.

Further advancing this research direction, Zhou et al. [12] developed a time-varying online transfer learning (TVOTL) model for intelligent fault diagnosis of rolling bearings. This model not only constructs a transfer diagnosis model offline using incomplete and unlabeled target data but also enables dynamic updates during online applications. This effectively addresses the challenge of acquiring complete data for all fault types during the model training phase.

Moreover, to overcome the limitations of current neural network-based fault diagnosis models—namely, limited depth, insufficient feature extraction capabilities, lack of adaptability, and poor classification performance across different domains—Liu et al. [13] proposed a novel fault prediction algorithm: the deep adaption residual neural network (DARN). Their research combines time-frequency representations, obtained by processing raw time-series vibration signals, with a DARN architecture incorporating a pre-trained model to enhance fault diagnosis capabilities. This pre-training approach reduces model complexity while simultaneously achieving superior performance, serving as a valuable demonstration of this methodology.

## 3. Research Method

### 3.1. Design of Experiments

To investigate fault diagnosis model generalizability, we developed an experimental protocol involving four distinct servo press models (OCP-110, G2-110, G2-160, and ST1-110), each subjected to nine operational conditions (Table 1 and Table 2) with three clearance levels, resulting in 27 experiments per model. Each experiment involved 3 h of operation, during which vibration signals were captured by two accelerometers at different locations. These signals underwent preprocessing, including data segmentation and transformation into spectrograms.

To simulate wear and tear, three clearance levels were established: standard clearance (normal operation), twice the standard clearance (mildly faulty), and three times the standard clearance (severely faulty), as determined through consultation with experienced maintenance engineers. Each press was also configured with three different stroke rates (SPM) and stamping intensities.

The four selected press models represent diverse mechanical designs. The OCP-110 utilizes a traditional C-frame, single-crank configuration, offering simplicity and rigidity at a low cost. The G2 models (G2-110 and G2-160) incorporate a C-frame with a double-crank mechanism, enhancing stability. The ST1-110 employs a modern straight-column, single-crank architecture, providing increased rigidity and precision at a higher cost. The numerical suffixes (110 and 160) denote press capacities in tons.

This model selection allows for evaluating the impact of structural design on generalization performance, specifically comparing generalization between presses sharing a single-crank design (ST1-110 vs. OCP-110) and those sharing a C-frame structure (OCP-110 vs. G2-110/160). This comparative analysis will elucidate the influence of structural design and press capacity on model generalization effectiveness.

Initially, a baseline model was trained using data acquired from the OCP-110 press. Four CNN architectures were used to perform intra-machine model generalization, that is, to evaluate the model’s performance across different operating conditions within the same press model. These architectures included two custom-designed CNNs (CNN-10 Layer and CNN-Res) and two well-established architectures: Visual Geometric Group (VGG) 16 [14] and ResNet50 [15].

Following intra-machine model generalization, the model trained on the OCP-110 data was applied to two other press models, namely G2-110 and G2-160, to evaluate its inter-machine generalizability. The same four CNN architectures were utilized to assess the model’s generalizability across different press models.

Finally, the three best-performing models from the inter-model generalization phase were selected and further evaluated for their ability to generalize to the ST1-110 press.

#### 3.1.1. Signal Acquisition

The press equipment utilized in this study consisted of commercially available servo presses manufactured by K Company in Taiwan, specifically the OCP-110, G2-110, G2-160, and ST1-110 models. Vibration data were acquired using Tongtai smart sensors (model: TiACC-01, TONGTAI MACHINE & TOOL CO., LTD., Kaohsiung, Taiwan) with a sampling rate of 6.4 kHz. Each experimental condition was monitored for a minimum duration of 3 h.

**Figure 2:**
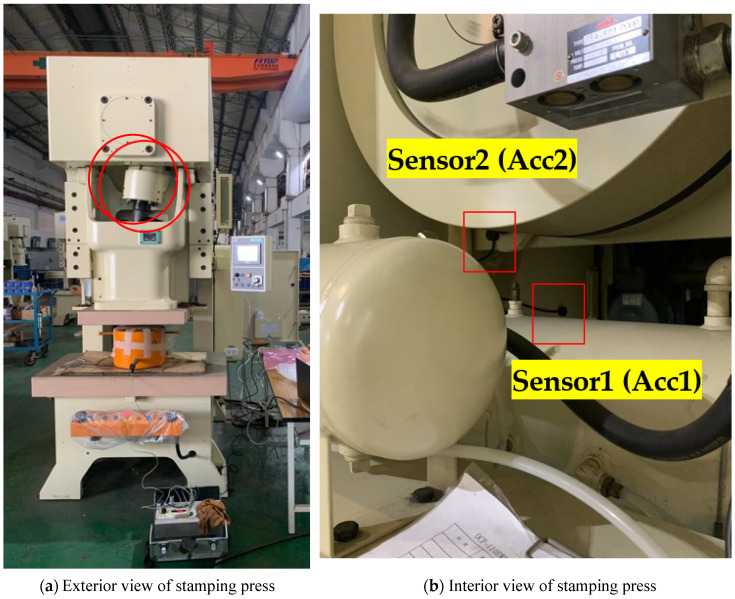
Sensor placement: (**a**) exterior view and (**b**) interior view.

To determine the optimal sensor placement, we consulted press experts. Based on their recommendations, two vibration sensors were installed: one (Acc1) on the vertical wall of the main inner press structure and the other (Acc2) on the flywheel support bracket, as depicted in Figure 2. Figure 2a shows the sensor placement from the exterior of the press, while Figure 2b provides an interior view.

**Figure 3:**
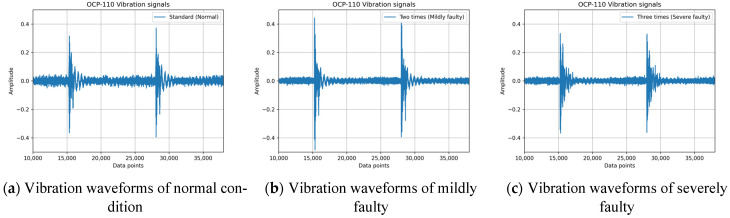
Vibration waveforms for an OCP-110 stamping press under three clearance conditions: (**a**) normal clearance (normal); (**b**) twice the normal clearance (mildly faulty); and (**c**) three times the normal clearance (severely faulty).

Following data preprocessing, the number of samples collected for each operating condition ranged from 500 to 800. This variation in sample size is attributed to differences in the configured strokes per minute (SPM) for each operating condition, despite a consistent data acquisition period of 3 h for each experiment, and each sample containing 60 cycles of vibration signal data. Figure 3 presents the vibration waveforms for an OCP-110 stamping press under three clearance conditions: normal clearance (normal), twice the normal clearance (mildly faulty), and three times the normal clearance (severely faulty). It is evident that three times the normal clearance correlates with increased instability in the vibration waveform, as shown in Figure 3c. From a practical operational perspective, the normal clearance represents the manufacturer’s default setting for a new press. Over time, as the press is used, the clearance tends to increase. Typically, when the press clearance exceeds three times the normal value, the quality of the stamped products becomes unacceptable, necessitating intervention from maintenance engineers to adjust the clearance and prevent excessive scrap rates.

#### 3.1.2. Data Preprocessing

In this study, continuous vibration signals, as depicted in Figure 1 and Figure 3, were subjected to preprocessing, which involved segmenting the data into smaller segments and applying the short-time Fourier transform (STFT) to convert the time-domain vibration signals into spectrograms [16]. As illustrated in Figure 3a, the horizontal and vertical axes of the spectrogram represent time and frequency, respectively. With prolonged press operation, the cumulative clearance gradually increases, leading to a decline in stamping quality and the corresponding changes in vibration frequencies. The conventional fast Fourier transform (FFT) is inadequate for capturing these temporal variations in frequency, thereby necessitating the use of an analysis method that provides both time and frequency information.

**Figure 4:**
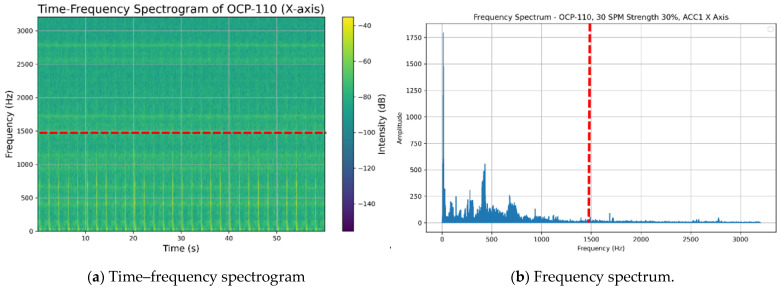
(**a**) Time–frequency spectrogram; (**b**) frequency spectrum.

To address this limitation, the STFT employs a sliding window function to segment the signal in the time domain, subjecting each segment to a Fourier transform. This approach allows for the capture of frequency variations at different points in time, thereby generating a time–frequency spectrogram that contains both time and frequency information, as depicted in Figure 4a. The STFT is mathematically defined as follows:

(1) $$S\left(f,k\right)=\sum_{n=0}^{N-1}S\left(n\right)\left[W\left(n-k\right)e^{-j^{2\pi fn/N}}\right]$$

where $S\left(n\right)$ represents the vibration signal in the time domain, f denotes frequency, and $W\left(n-k\right)$ is the Hamming window function.

Furthermore, an analysis of the time–frequency spectrogram, depicted in Figure 4a, revealed that the predominant characteristic signals of the punch press vibration are concentrated within the low-frequency range. Consequently, we examined the frequency spectrum above 1500 Hz and calculated the energy of the frequency band between 1500 Hz and 3200 Hz. This high-frequency band accounted for only 3.8% of the total energy across all frequencies, as illustrated in Figure 4b.

Based on these observations, we restricted the analysis frequency range to 0–1500 Hz, effectively filtering out frequency signals above 1500 Hz. This approach facilitated the extraction of pertinent features for subsequent analyses. The proposed data preprocessing strategy not only enhanced the efficiency of model training but also established a foundation for improving the generalization performance of the fault diagnosis models.

#### 3.1.3. Implementation of Generalized Fault Diagnosis Models for Punching Machines

To develop predictive models for differentiating faults, specifically varying clearances in punching machines, the time–frequency images generated through preprocessing were input into deep learning algorithms. Four deep learning models were used to investigate the generalization performance for fault classification: CNN-10 Layers, CNN-Res, VGG16, and ResNet50.

VGG is a deep convolutional neural network architecture [14] developed by the Visual Geometry Group at the University of Oxford, and this architecture is renowned for its straightforward structure and exceptional performance. We used a VGG network pretrained on a dataset from the OCP-110 machine, which was then transferred to other machines. VGG is composed primarily of stacks of small 3 × 3 convolutional kernels and 2 × 2 pooling kernels that form a deep network structure. This streamlined design facilitates comprehension, implementation, modification, and expansion. By stacking multiple small convolutional kernels, VGG effectively increases network depth, thereby enhancing the model’s nonlinear expressive capacity and allowing it to learn more intricate image features. Compared to networks that employ larger convolutional kernels, VGG requires fewer parameters and exhibits higher computational efficiency at the same depth.

However, VGG16 contains a substantial number of parameters (approximately 138 M), and therefore, it needs more storage space and computational resources. Consequently, model training times are prolonged and overfitting may occur. Moreover, owing to its depth, VGG demands considerable computational power, requiring powerful GPUs for training and inference.

**Figure 5:**
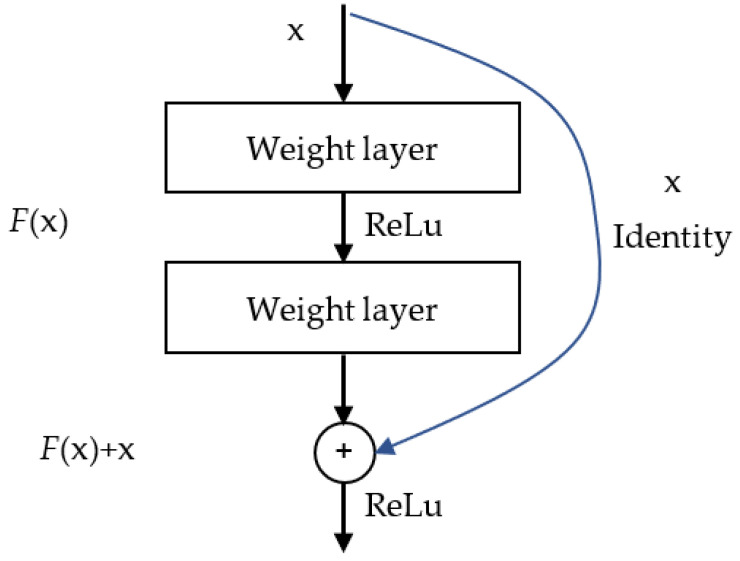
Residual learning: a building block [15].

ResNet50, a variant of the deep residual network (ResNet) introduced by He et al. [15] at Microsoft Research, is a widely used CNN architecture that has been remarkably successful in image recognition tasks. The “50” in ResNet50 denotes the 50 layers comprising the network. As illustrated in Figure 5, the core concept underlying ResNet is the introduction of “a deep residual learning framework” to address the degradation problem commonly encountered in deep networks. Traditional deep neural networks suffer from vanishing or exploding gradients as the number of layers increases, which hinders training. ResNet’s key innovation lies in employing “residual learning” by incorporating “skip connections” between every few layers. These connections directly transmit the input to the subsequent layers, which mitigates the vanishing gradient problem.

Specifically, ResNet50 consists of 48 convolutional layers, one max-pooling layer, and one fully connected layer. The entire model is constructed by stacking multiple residual blocks, each typically containing two or three convolutional layers. These “skip connections” allow data to bypass certain layers and be passed directly to deeper layers.

A key advantage of ResNet50 is its effective balance between depth and performance. Compared to shallower networks, it can learn more complex features while maintaining a high training speed and excellent performance owing to the residual blocks. Despite being deeper than networks such as VGG16, ResNet50 has a relatively lower parameter count because of its residual block design. In this study, a ResNet50 network pretrained on an OCP-110 machine dataset was used and subsequently transferred to other machines.

In traditional CNNs, the network learns a mapping $H\left(x\right)\;$ directly from the input x. However, the introduction of residual blocks modifies this mapping to learn a residual function $F\left(x\right):=H\left(x\right)-x$. Consequently, the final output y can be expressed as follows:

(2) $$y=F\left(x\right)+x$$

where $F\left(x\right)$ represents the residual function learned through a series of convolutional layers. This design allows the input x to be propagated directly to the output, even when the learned residual function $F\left(x\right)$ is weak, which effectively mitigates the vanishing gradient problem. By adding the identity mapping x to the output of the residual function, the network can learn to approximate the residual mapping, which is often easier than learning the original mapping directly. This approach facilitates the training of deeper networks and improves overall performance.

To address the high computational demands of VGG16 and ResNet50, a customized, lightweight CNN architecture—CNN-10 Layers—was developed in this study. The primary objective of this design was to maintain satisfactory image classification performance while reducing computational resource requirements, thereby enabling deployment on the industrial computers that are typically used in punching machines.

Industrial computers often have limited processing power and memory. Deploying computationally intensive deep learning models, such as VGG16, on such hardware can hinder real-time computation. To overcome this constraint and facilitate deployment on resource-constrained devices, CNN-10 Layers was derived from VGG16 through simplification. Specifically, the first, second, and last convolutional blocks were modified. The original structure, consisting of two convolutional layers followed by max-pooling, was reduced to a single convolutional layer followed by max-pooling. Additionally, sections containing three consecutive convolutional layers were removed entirely. These modifications considerably reduced the number of convolutional and fully connected layers, thereby decreasing the model’s parameter count and computational cost.

**Figure 6:**
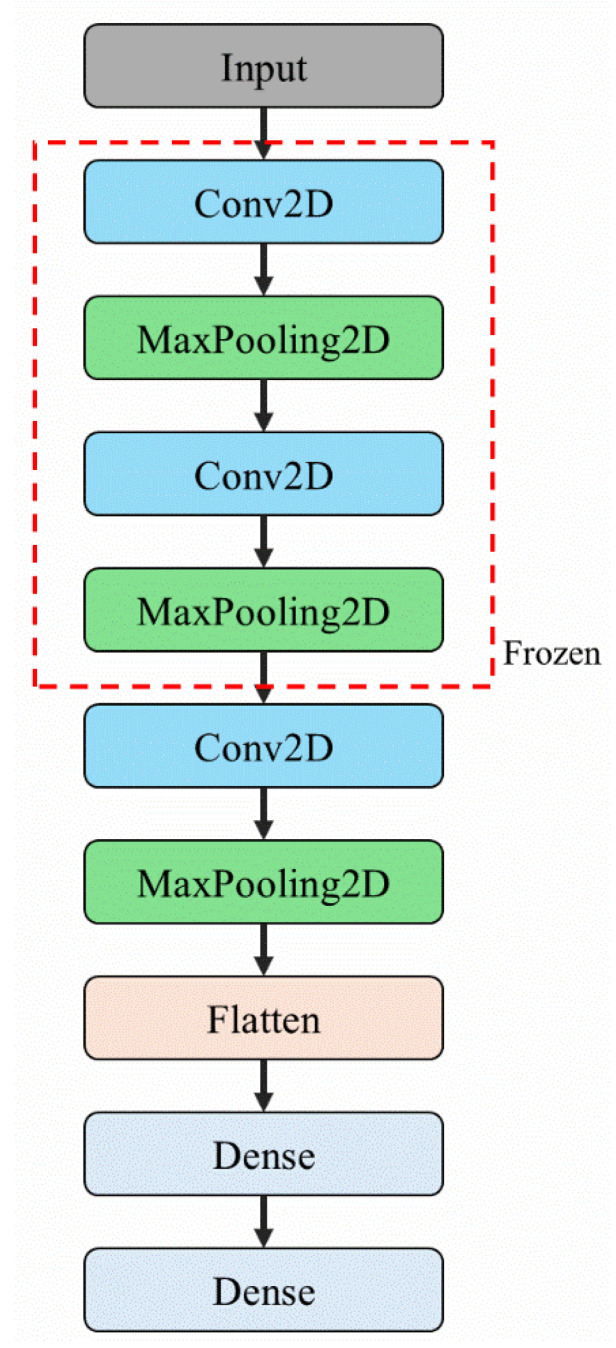
Schematic overview of the CNN-10 Layers architecture.

Despite its reduced depth, CNN-10 Layers retains the core design principle of VGG16, utilizing small 3 × 3 convolutional kernels for feature extraction across layers. This modification aims to effectively preserve the efficient learning of local image features while streamlining the model architecture. Figure 6 schematically illustrates the simplification process followed to develop CNN-10 Layers.

In addition to CNN-10 Layers, a custom deep learning architecture, CNN-Res, was developed. CNN-Res integrates the residual block concept from ResNet50 into the CNN-10 Layers architecture. By incorporating two ResNet50 residual blocks, the model’s performance and stability were further enhanced.

This design leverages the simplicity and computational efficiency of CNN-10 Layers while addressing the vanishing or exploding gradient problem often encountered in deep networks through the use of ResNet50′s residual learning mechanism. Specifically, the CNN-Res model introduces a residual block following the first set of convolutional and max-pooling layers in CNN-10 Layers, and another following the second set. To accommodate this added complexity, one set of convolutional and max-pooling layers is removed.

**Figure 7:**
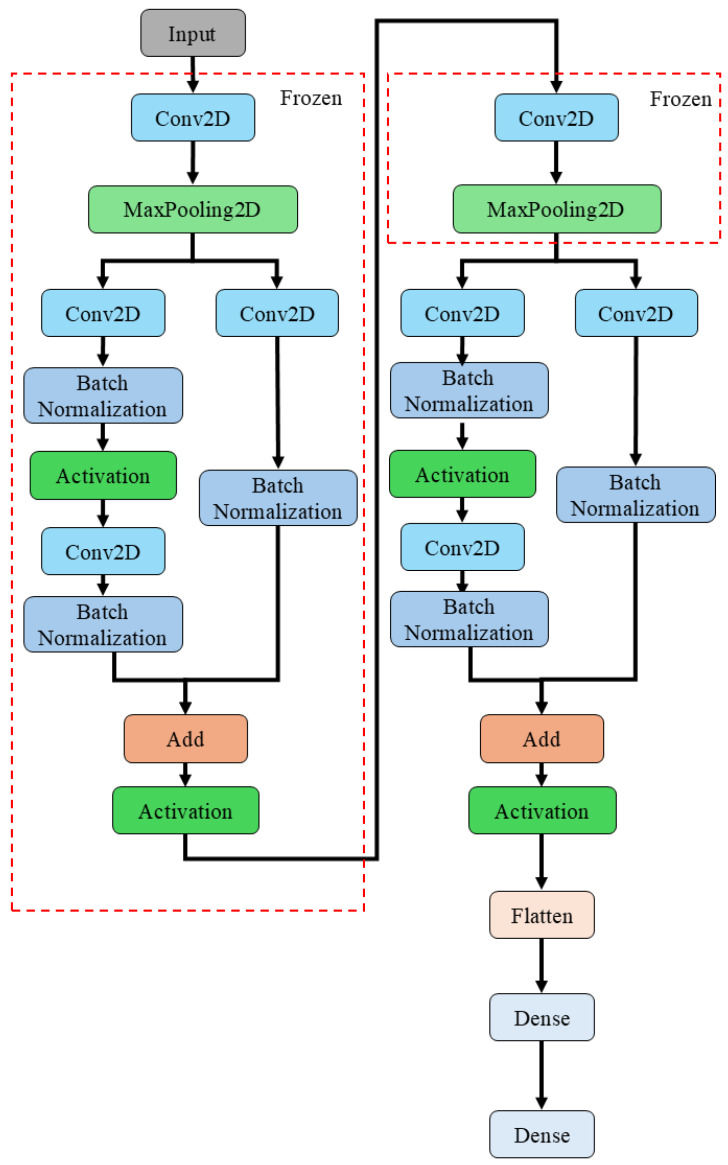
Schematic overview of CNN-Res architecture development.

The CNN-Res architecture improves the network’s ability to learn from more complex data while maintaining computational efficiency. The residual blocks allow the input to bypass certain convolutional layers and be added directly to the output, thereby mitigating the vanishing gradient problem and enhancing the training process. Figure 7 schematically depicts the architecture simplification process.

Model training in this study was conducted using the following hardware configuration: an Intel Core i3-9100F CPU; 24 GB of RAM; and an NVIDIA GeForce GTX 1060 (6 GB) GPU. The software environment comprised Python 3.9.19, TensorFlow-gpu 2.9.0, CUDA 11.2, and cuDNN 8.2.0.

### 3.2. Transfer Learning

Transfer learning leverages the knowledge embedded within a pretrained model to expedite and enhance the learning process for new tasks, leading to accelerated training and improved performance [17,18]. Its primary objective is to improve model performance in the target domain by utilizing the knowledge acquired from the source domain. Specifically, a model pretrained on the source domain learns a set of feature representations that can be transferred efficiently to the target domain, which facilitates faster model convergence and enhances performance on the new task.

In this study, transfer learning plays a pivotal role in the development of a generalized fault diagnosis model. The process consists of the following progressive steps:

1. Baseline model training: initial training is conducted on three operating conditions of the OCP-110 machine.
2. Intra-machine generalization: the trained models are then extended to other operating conditions within the same OCP-110 machine.
3. Inter-machine generalization (similar conditions): the models are further generalized to different machine types, G2-160 and G2-110, under similar operating conditions.
4. Cross-machine and cross-condition generalization: finally, generalization is extended to the ST1-110 machine, which has different conditions.

### 3.3. Fine-Tuning Strategy

The vibration signals generated by different punching machine models exhibit inherent variations owing to differences in operating conditions, structural characteristics, and other factors. These variations result in distinct signal distributions, as illustrated in Figure 1. The signals from different machines may contain unique features within specific frequency bands, reflecting the disparities in their underlying distributions.

Consequently, to develop effective fine-tuning strategies for transfer learning in this context, two primary approaches were explored with a focus on adapting the model to the distinct vibration signal characteristics of each machine to achieve optimal generalization performance [19]. This investigation aimed to assess the impacts of different fine-tuning strategies on the performance of the generalized fault diagnosis models.

1. Full-layer fine-tuning (FLF): This approach involves retraining all layers of the pretrained model without freezing any parameters. By allowing the model to fully adjust to the new machine’s data, FLF facilitates the learning of novel features, which is particularly beneficial when the new machine exhibits significant differences in operational characteristics compared to the original machine. However, this method requires extended training times and substantial amounts of labeled data to ensure robust model adaptation.
2. Partial-layer fine-tuning (PLF): In this approach, a portion of the pre-trained model’s layers—typically half—are frozen while the remaining layers are fine-tuned. As illustrated in Figure 6 and Figure 7, the segments enclosed within the red dashed rectangles represent the frozen layers. During the fine-tuning process, the weights of these layers are held constant, thereby preserving the generalized features acquired during the pre-transfer learning phase. Conversely, the layers situated outside the red rectangles are permitted to undergo parameter updates, enabling the model to capture features relevant to the novel target task based on the new machine-specific data. By preserving the fundamental features learned from the OCP-110 data, PLF allows the model to adapt to new vibration signal characteristics while maintaining the knowledge acquired from the source domain. This strategy is particularly suitable when the new machine’s data shares structural similarities to the original dataset. Additionally, PLF reduces training time and mitigates the risk of catastrophic forgetting by preventing excessive modification of the pre-trained weights.

Vibration signals from different machine models can vary considerably, necessitating fine-tuning to enhance the adaptability of a pretrained model to new data distributions.

In this study, a baseline model was initially trained using the spectrograms derived from the OCP-110 punching machine. This model was then generalized to other punching machine models. The generalization performance of the baseline model was evaluated under two distinct fine-tuning strategies: FLF and PLF. This analysis provided insights into how different adaptation approaches influence the model’s ability to classify faults effectively across diverse machine types.

### 3.4. Process of Building a Generalized Fault Classification Model for Punch Presses

To address the challenge of generalization in fault classification models for servo presses, this study presents a framework for constructing optimal generalized models. This framework was validated through extensive experimentation, resulting in a fault diagnosis model capable of handling diverse machine types and operating conditions.

**Figure 8:**
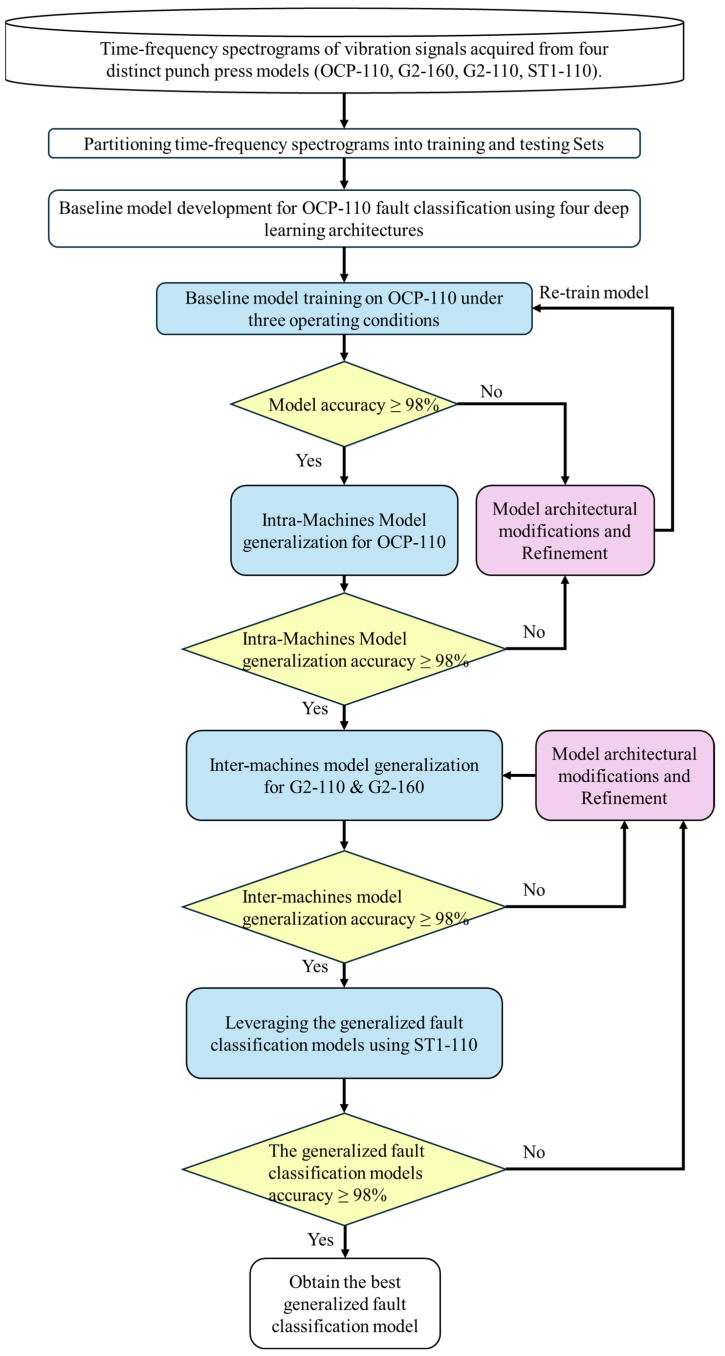
Process of building a generalized fault classification model for punch presses.

As depicted in Figure 8, the process commenced with vibration signal acquisition from four punching machine models (OCP-110, G2-160, G2-110, ST1-110) by using two accelerometers (Acc1 and Acc2) positioned at different locations on each machine. Time-domain signals were transformed into spectrograms using a STFT to capture time-varying frequency characteristics. Then, the processed time–frequency spectrograms were randomly partitioned into training and testing sets in an 8:2 ratio. The fault classification models were then developed using the training data of the OCP-110 machine and employing four deep learning architectures: VGG16, ResNet50, CNN-10 Layers, and CNN-Res.

Initially, three distinct operating conditions were selected, as outlined in Table 3:

- Condition 1: 30 SPM and 30% stamping intensity;
- Condition 2: 45 SPM and 60% stamping intensity;
- Condition 3: 60 SPM and 90% stamping intensity.

These conditions were chosen strategically to cover a wide range of practical operating scenarios by varying stamping speeds and intensities. For instance, condition 1 represents low speed and low intensity, condition 2 represents medium speed and medium intensity, and condition 3 represents high speed and high intensity. The models were trained and evaluated under these conditions with a minimum accuracy of 98% required before proceeding to model generalization. This stringent performance threshold ensured model robustness and reliability before extending their application to other machines and operating conditions.

To assess intra-machine generalization, baseline models trained on the three operating conditions of the OCP-110 were applied to additional conditions within the same machine. Models achieving a minimum accuracy of 98% were advanced to inter-machine generalization, while those failing to meet this threshold underwent iterative adjustments and retraining.

Inter-machine generalization involved adapting the models to different machine types (G2-160 and G2-110) using transfer learning with two fine-tuning strategies: FLF and PLF. Models achieving less than 98% accuracy were deemed inadequate for effective fault diagnosis on the new machines.

Finally, to assess generalization across different machine types and operating conditions, an additional evaluation was conducted on the ST1-110 machine with varying stamping intensities (60–90%), as outlined in Table 2.

## 4. Results and Discussion

### 4.1. Evaluation of Baseline Models

To evaluate the performance of the fault diagnosis model, the following metrics were employed: accuracy, recall rate, and the F1-score. These are defined as follows:

(3) $$Accuracy=\frac{TP+TN}{TP+FP+FN+TN}$$

(4) $$Recall\;rate=\frac{TP}{TP+FN}$$

(5) $$F1\;score=\frac{2\times Presion\;rate\times Recall\;rate}{Presion\;rate+Recall\;rate}$$

where *TP*, *TN*, *FP*, and *FN* represent the number of true positives, true negatives, false positives, and false negatives, respectively, as derived from the confusion matrix. To mitigate the risk of model overfitting, a ten-fold cross-validation procedure was implemented to evaluate model performance. The primary objective during hyperparameter optimization was the maximization of accuracy, recall rate, and the F1-score.

Analysis of the results indicated that four distinct convolutional neural network (CNN) architectures—a custom 10-layer CNN (CNN-10 Layers), a custom residual network (CNN-Res), VGG16, and ResNet50—were employed to train baseline models using data acquired from the OCP-110 machine. Training was performed under three distinct operating conditions, defined by combinations of strokes per minute (SPM) and stamping intensity: (30 SPM, 30% intensity), (45 SPM, 60% intensity), and (60 SPM, 90% intensity). This process yielded a total of 12 baseline models (Models I-XII), as summarized in Table 4. All twelve baseline models demonstrated accuracy exceeding 98%. The average accuracy, recall, and F1-score across these models were 99.57% ± 0.60% (Acc1) and 99.47% ± 0.65% (Acc2); 99.44% ± 0.45% (Acc1) and 99.14% ± 0.83% (Acc2); and 99.49% ± 0.46% (Acc1) and 99.26% ± 0.66% (Acc2), respectively.

In summary, models I–XII consistently achieved accuracies, recall rates, and F1 scores of 98% or higher across all combinations of low, medium, and high stamping speeds and intensities, with many conditions achieving 100% accuracy. Therefore, the employed CNN architectures possessed robust and effective fault classification capabilities across a wide range of operating conditions, adapting to varying stamping speeds and intensities while maintaining high accuracy. These findings provided strong evidence for the reliability and suitability of these architectures for fault classification applications in dynamic operational environments.

Based on these promising results, the next step involved generalizing these baseline models, trained on the three selected operating conditions, to other operating conditions within the OCP-110 machine to further investigate their generalization capabilities.

### 4.2. Constructing Generalization Model Without Transfer Learning

Initially, we employed baseline model I, a CNN-10 Layers architecture trained on an operational condition of 30 SPM with a stamping intensity of 30%, to predict failures in the OCP-110 press under eight other operating conditions. The results, presented in Table 5, demonstrate a significant lack of predictive performance. The model’s highest accuracy, recall rate, and F1-score all failed to exceed 75%. This clearly indicates that the baseline model, trained on a single operational condition, cannot effectively generalize to predict failures under different conditions. Therefore, to achieve the objective of model generalization, we must explore the application of transfer learning techniques.

### 4.3. Intra-Machine Fault Classification Generalization Model

In this section, we evaluate the generalization performance of the baseline models trained on different CNN architectures within the OCP-110 machine. This analysis focuses on the ability of these models, trained on specific operating conditions, to generalize across other conditions within the same machine. The results are shown in Table 6.

Baseline model I, as an example (trained with CNN-10 Layers architecture at 30 SPM and 30% stamping intensity), was generalized to other conditions by applying fine-tuning strategy PLF. The performance of these generalized models was assessed using accelerometers Acc1 to identify the potential variations in accuracy between the sensors. The results indicate that the generalized model, when trained with partial-layer fine-tuning (PLF) and the Acc1 dataset, achieved an average accuracy, recall, and F1-score of 98.64%, 98.63%, and 98.65%, respectively. Employing the CNN-Res architecture (corresponding to baseline model II) yielded improved performance, with an average accuracy, recall, and F1-score of 99.53%, 99.57%, and 99.55%, respectively. Therefore, the generalization performance of baseline model II demonstrably surpassed that of baseline Model I.

Averaging the performance metrics across all twelve baseline models revealed consistently high performance, exceeding 99%, irrespective of the fine-tuning strategy employed (partial-layer fine-tuning (PLF) or full-layer fine-tuning (FLF)) and the dataset used (Acc1 or Acc2). These results, summarized in Table 5, demonstrate the excellent performance of the CNN-based intra-machine fault classification generalization model. These consistently high accuracies demonstrate the exceptional generalization capabilities of these architectures, underscoring their robustness and adaptability in handling varying operating conditions within a single machine. This ensures effective fault diagnosis across a range of scenarios.

A comparison of Table 5 and Table 6 reveals a significant improvement in the performance of the generalized model when employing transfer learning, irrespective of whether the fine-tuning strategy used is PLF or FLF. The accuracy, recall rate, and F1-score achieved with transfer learning substantially surpassed those obtained without it. This observation strongly suggests that the utilization of transfer learning is crucial for achieving superior performance in model generalization.

### 4.4. Generalization of Fault Classification Models Across Different Machines

Having successfully generalized the baseline fault classification models to various operating conditions of the OCP-110 machine, we investigated the feasibility of extending this generalization to different punching machine models: G2-110 and G2-160. As indicated by their model designations, the G2-110 has a maximum stamping intensity of 110 tons, while the G2-160 has a maximum stamping intensity of 160 tons.

In this experiment, both G2-110 and G2-160 were operated under different operational conditions, including stamping speeds of 30 SPM, 45 SPM, and 60 SPM, combined with stamping intensities of 30%, 60%, and 90%, resulting in nine distinct operating conditions for each machine. This diverse set of conditions allowed for a comprehensive evaluation of the models’ adaptability to varying machine characteristics and operating parameters. Both the PLF and FLF methods were employed, and the results obtained at both accelerometer locations (Acc1 and Acc2) were analyzed.

The results presented in Table 7 demonstrate that the inter-machine fault classification generalization models for the G2-110 press exhibited excellent generalization performance. Specifically, the generalized model, trained with partial-layer fine-tuning (PLF) and the Acc1 dataset, achieved an average accuracy, recall, and F1-score of 98.73%, 98.71%, and 98.73%, respectively. Furthermore, employing the CNN-Res architecture (corresponding to baseline model II) yielded superior performance, with an average accuracy, recall, and F1-score of 99.93%, 99.90%, and 99.92%, respectively. Consequently, the generalization performance of baseline model II demonstrably surpassed that of baseline model I.

Similar trends are evident in Table 8. Averaging the performance metrics across all twelve baseline models revealed consistently high accuracy, recall, and F1-scores, exceeding 99% for both the G2-110 and G2-160 presses, irrespective of the fine-tuning strategy employed (partial-layer fine-tuning (PLF) or full-layer fine-tuning (FLF)) and the dataset used (Acc1 or Acc2).

An analysis of the results presented in Table 7 and Table 8 provided valuable insights into the inter-machine generalization performance of the various CNN architectures. Among the baseline models trained on the OCP-110 machine, CNN-Res and VGG16 exhibited the best overall performance, followed by ResNet50, with the CNN-10 Layers architecture demonstrating the weakest generalization capability.

The overall average accuracies of CNN-Res, VGG16, and ResNet50 exceeded 99%, highlighting their strong generalization abilities across different machine types and operating conditions. By contrast, the CNN-10 Layers architecture achieved a lower overall average accuracy, falling below 99%. This suggested that the deeper and more complex architectures of CNN-Res, VGG16, and ResNet50 were more suitable for capturing and transferring the features relevant to fault diagnosis across various machines.

Furthermore, the results consistently demonstrate that both accelerometer locations (Acc1 and Acc2) provided effective signals for successful model generalization. This indicates that the accelerometer placement at these two locations adequately captured the necessary vibration data for fault diagnosis, and the choice of sensor location did not significantly influence the generalization performance.

Interestingly, the choice of deep learning architecture and fine-tuning strategy (PLF vs. FLF) notably affected the generalization performance. This finding warrants further investigation to understand the underlying mechanisms and optimize the selection of these factors for achieving optimal generalization in fault diagnosis applications. Future research could explore the effects of different architectural components, such as the number and type of layers, on generalization performance. Additionally, a more detailed analysis of the effects of different fine-tuning strategies could provide valuable insights into improving the adaptability of fault diagnosis models across diverse machine types and operating conditions.

## 5. Selection and Validation of Optimal Generalized Model

### 5.1. Comparative Analysis of Inter-Machine Generalization Performance Across Four CNN Architectures

In this section, we focus on the selection and validation of the optimal generalized model for fault diagnosis in punching machines based on the comprehensive analysis conducted in the previous sections. In the selection process, we consider the performances of the different CNN architectures across machine types and operating conditions to identify a model that has robust generalization capabilities and high fault classification accuracy.

Table 8 provides a comparative analysis of the cross-machine generalization performances of the four CNN architectures (CNN-10 Layers, CNN-Res, VGG16, and ResNet50) across three punching machine models: OCP-110, G2-110, and G2-160. The analysis assesses the relative strengths and weaknesses of each architecture in adapting to different machine types and operating conditions, ultimately guiding the selection of the optimal architecture for generalized fault diagnosis.

To assess the generalization performance of the baseline models (models I–XII) across different machine types and operating conditions, the accuracy of each model was averaged across 27 generalization scenarios, which included three machine types (OCP-110, G2-110, and G2-160) and nine operating conditions per machine. This approach allowed us to identify the models exhibiting superior stability across diverse conditions, in addition to highlighting specific model–condition combinations that yielded exceptional performance.

By quantifying the variability of predictive performance across different operating conditions, this evaluation method facilitates the optimization of model application strategies. It ensures overall classification accuracy and practicality by identifying the models that consistently perform well across diverse scenarios.

Furthermore, this method offers an intuitive assessment of the predictive capabilities of models I–XII, as summarized in Table 8. The analysis allows us to select the optimal model or combination of models capable of generalizing effectively across different punching machines and their diverse operating conditions. This rigorous evaluation process guarantees the identification of robust and reliable models for real-world deployment in fault diagnosis applications.

According to Table 9, the model combination with the highest average accuracy was baseline model II, which used the FLF strategy and achieved an average accuracy of 99.88% ± 0.19%. This suggests that with this specific configuration, the model’s predictive accuracy approaches the ideal state. These findings indicate that the optimal model stability and generalization are achieved when the baseline model is trained under the following conditions: (1) an operating condition of 30 SPM with 30% stamping intensity; (2) the CNN-Res CNN architectureCNN-Res; (3) the FLF fine-tuning strategy; (4) Acc2 sensor placement, positioned on the flat surface of the flywheel.

The second-best performing model was also baseline model II but with the PLF strategy, and it achieved an average accuracy of 99.87% ± 0.22%. The third-best model was baseline model VI, which utilized the CNN-Res architecture and FLF strategy but was trained on data acquired from accelerometer Acc1, positioned on the vertical wall of the main internal structure of the punching machine. It achieved an average accuracy of 99.87% ± 0.21%.

The above analysis highlights the importance of carefully selecting the operating conditions, CNN architecture, fine-tuning strategy, and sensor placement when developing generalized fault diagnosis models with high accuracy and robustness.

### 5.2. Validation of Optimal Generalized Model Using ST1-110 Press Machine

The validation process involved rigorous testing of the selected model on unseen data to confirm its effectiveness and reliability in real-world scenarios. To further validate the three optimal model combinations identified in the previous section, experiments were conducted using the data collected from the ST1-110 machine. The experimental parameters of ST1-110 are listed in Table 2.

In the ST1-110 validation experiments, the stamping intensity was varied to simulate changes in production load, which helped examine the models’ performance stability under diverse operating conditions and across different servo press models. This approach aimed to evaluate the models’ adaptability to varying loads and different servo press characteristics while also assessing their generalization capabilities in practical applications.

Based on the three optimal model combinations identified earlier, we further evaluated their performance in fault classification with a focus on accuracy and stability under varying loads. Additionally, model computation time was included as another performance evaluation metric. The results of this model generalization validation are presented in Table 10.

An analysis of the average prediction accuracy values listed in Table 10 revealed that model VI, utilizing FLF and accelerometer Acc1, achieved the highest accuracy of 99.76% ± 0.50%. The second-best-performing model was model II, employing PLF and accelerometer Acc2, with an accuracy of 99.56% ± 0.87%. Model II with FLF and Acc2 yielded the lowest accuracy of 99.32% ± 0.84% among the three.

However, when considering the practical requirements of a manufacturing production line, factors beyond high accuracy, such as retraining time for generalized models and preservation of features from the base model, become critical. Upon further examination of Table 9, model VI with FLF and Acc1 showed only a 0.2% difference in accuracy compared to model II with PLF and Acc2, but it required approximately 3.6 times longer for retraining. Therefore, in view of the needs of production lines, we selected model II with PLF and Acc2 as the optimal generalized model because of its balance of high accuracy and efficient retraining time.

### 5.3. Comparison of the Learning Process Between the Pre-Fine-Tuning and the Post-Fine-Tuning

**Figure 9:**
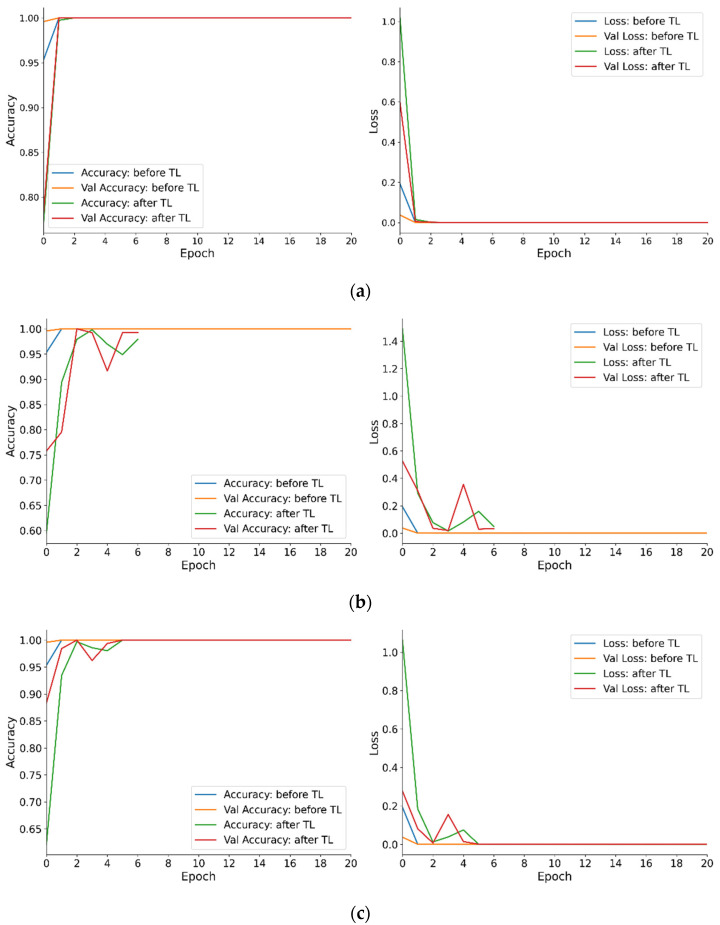

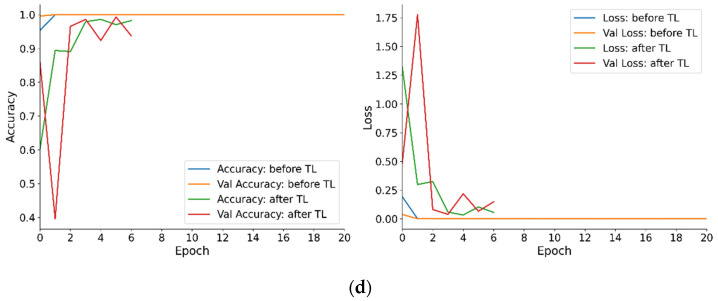
Models’ learning process between the pre-fine-tuning and post-fine-tuning with model II: (**a**) OCP-110, 30 SPM and 60%; (**b**) G2-110, 60 SPM and 60%; (**c**) G2-160, 45 SPM and 60%; and (**d**) ST1-110, 60 SPM and 90%.

In Figure 9, we present the transfer learning process using baseline model II to generalize to different machine types and operational conditions. The learning curves in Figure 9a–d illustrate the trends of accuracy and loss during the training process before and after transfer learning. Observable from these curves is the gradual increase in accuracy and eventual plateauing after a certain number of epochs, concurrent with a sustained decrease in loss, demonstrating effective model learning and convergence. To mitigate overfitting, an early stopping mechanism with a patience parameter of five was implemented, which terminates iterative computation if the validation loss fails to improve for five consecutive epochs. The Adam optimizer was employed with a learning rate of 0.001 and a batch size of eight. The results indicate that the pre-fine-tuning model rapidly captured fundamental features, while the post-fine-tuning model further refined parameters for the target task, ultimately achieving stable convergence. The comparative analysis of accuracy and loss curves reveals minimal divergence between training and validation curves, suggesting the absence of significant overfitting. The early stopping mechanism is triggered upon cessation of validation accuracy improvement or loss reduction, thereby preventing unnecessary iterations and ensuring model generalization.

### 5.4. Assessment of the Generalizability of the Model Using Pre-Transfer Learning Dataset

In this study, three operational conditions of the OCP-110 press model were utilized as the pre-transfer learning dataset to establish the baseline model, as detailed in Table 3. Subsequently, transfer learning and fine-tuning strategies were employed to apply the model to different machines and operational conditions, namely G2-110, G2-160, and ST1-110. As evidenced by the results presented in Table 6, Table 7 and Table 8, the average accuracy, recall rate, and F1 score achieved by the four CNN architectures, in conjunction with transfer learning and fine-tuning, all surpassed 99%. However, in the realm of deep learning, ’catastrophic forgetting’ poses a significant challenge, wherein models tend to forget previously learned tasks when trained on new ones, which is particularly problematic for our objective of developing a generalized model capable of continuous learning across diverse machine types and operational conditions [20]. Therefore, to rigorously evaluate the performance of our selected optimal model, specifically model II with PLF and Acc2, the pre-transfer learning dataset was reintroduced for validation following the application of transfer learning and fine-tuning. The results revealed an average accuracy, recall rate, and F1 score of 99.90 ± 0.32, 99.90 ± 0.32, and 99.90 ± 0.33, respectively. This demonstrates that the fine-tuned model not only excels in learning across different machine types and operational conditions but also effectively retains the generalized features learned during the pre-transfer learning phase. Consequently, our model successfully preserves the source domain knowledge acquired from the pre-transfer learning dataset while seamlessly integrating new machine and operational condition-specific knowledge, thereby achieving efficient knowledge transfer.

## 6. Discussion

### 6.1. Scalability of Generalized Fault Classification Model

This study demonstrates the feasibility of a generalized fault classification model applicable to different press types, including C-frame and straight-column structures with varying capacities (110 and 160 tons). This model can also be applied across different manufacturer brands with similar press structures.

The selection of C-frame and straight-column presses is justified as they represent high-volume sales models and are favored by different enterprise scales due to their respective advantages in cost, rigidity, and automation compatibility. Furthermore, despite variations in structural design, the primary vibration sources in power presses remain consistent, suggesting the representative nature of the four machine models employed in this study.

While this study focuses on generalization across different machine types and operating conditions, future work will address the model’s scalability to a wider range of fault types, such as those caused by lubricant insufficiency or degradation.

### 6.2. Significance of Model Retraining Time in Industrial Applications

Model retraining time is crucial for practical deployment in industrial settings. While model VI with FLF and Acc1 achieved marginally higher generalization accuracy (0.2%) than model II with PLF and Acc2 (Table 9), its retraining time was significantly higher (3.6 times that of model II). In industrial applications, longer retraining times lead to increased downtime and reduced efficiency, negatively impacting production capacity. Therefore, prioritizing models with shorter retraining times, such as model II, is essential for minimizing disruptions and maintaining productivity. This highlights the critical trade-off between accuracy and efficiency in model selection for time-sensitive industrial environments.

## 7. Conclusions

In modern industries, maximizing equipment uptime, enhancing production capacity, minimizing downtime owing to equipment failures, and reducing repair time are critical factors for manufacturing success. To this end, the development of generalized fault diagnosis models that can be applied to a wide range of machine types and fault modes is a practical and effective solution. By proactively detecting potential failures and implementing predictive maintenance strategies, manufacturers can prevent unanticipated downtime, mitigate production losses, and maintain high operational efficiency.

Considering both overall accuracy and retraining time, model II, based on the custom CNN-Res architecture coupled with the PLF strategy and vibration signals from accelerometer Acc2, was identified as the optimal generalized model. Validation on a different punching machine model, ST1-110, yielded a retraining time of 523 s and an overall accuracy of 99.56% ± 0.87%, emphasizing the model’s outstanding generalization performance.

An analysis of four distinct deep learning architectures revealed that the number of layers significantly influenced generalization performance. For example, ResNet50, the deepest architecture tested herein, achieved good generalization with PLF, but its performance deteriorated, and its retraining time increased substantially when using FLF. This suggests that deeper architectures benefit from PLF to maintain efficient generalization.

By contrast, CNN-Res and VGG16, with moderate architectural depths, achieved excellent generalization performance with both PLF and FLF. This result highlights the importance of optimizing architectural depth to strike a balance between accuracy and efficiency. Further research is needed to systematically explore the relationship between architectural depth and generalization performance.

Overall, this study highlighted the effectiveness of CNN-based deep learning models, combined with different fine-tuning strategies, for generalized fault diagnosis in servo presses. The CNN-Res architecture with PLF consistently achieved high accuracy and low retraining time, outperforming other models in both intra-machine and inter-machine generalization. By preserving the features from the base model through a frozen-layer approach, CNN-Res with PLF optimally balanced accuracy and efficiency. Based on these findings, we recommend prioritizing CNN-Res with PLF as the preferred solution for generalized fault classification in punching machines in real-world industrial environments.

## Figures and Tables

**Table 1:** Experimental parameters of OCP-110, G2-160, and G2-110 [5].

Press Model	Stroke Rate (SPM)	Stamping Intensity (%)	Clearance and Fault Type
OCP-110	30, 45, 60	30, 60, 90	Standard (Normal)
G2-110			Two times (Mildly faulty)
G2-160			Three times (Severe faulty)

**Table 2:** Experimental parameters of ST1-110 [5].

Press Model	Stroke Rate (SPM)	Stamping Intensity (%)	Clearance and Fault Type
ST1-110	30, 45, 60	60, 75, 90	Standard (normal)
			Two times (mildly faulty)
			Three times (severely faulty)

**Table 3:** OCP-110 baseline model training: Three distinct operating conditions.

Operating Condition (OC)	Stroke Rate (SPM)	Stamping Intensity (%)	Clearance and Fault Type
1	30	30	Standard (normal)
2	45	60	Two times (mildly faulty)
3	60	90	Three times (severely faulty)

**Table 4:** Definition of the baseline model with various CNN architectures.

Baseline Model	CNN Architecture	Machine Model	SPM	Strength (%)
I	CNN-10 Layers	OCP-110	30	30
II	CNN-Res			
III	VGG16			
IV	ResNet50			
V	CNN-10 Layers	OCP-110	45	60
VI	CNN-Res			
VII	VGG16			
VIII	ResNet50			
IX	CNN-10 Layers	OCP-110	60	90
X	CNN-Res			
XI	VGG16			
XII	ResNet50			

**Table 5:** Performance evaluation of fault classification generalization models without transfer learning.

Operating Conditions	Acc1			Acc2		
	Accuracy	Recall	F1-Score	Accuracy	Recall	F1-Score
30 SPM/60%	66.57%	66.57%	66.04%	49.71%	49.71%	48.10%
30 SPM/90%	58.05%	58.05%	54.51%	46.70%	46.70%	47.49%
45 SPM/30%	72.12%	72.12%	71.89%	52.56%	52.56%	52.44%
45 SPM/60%	43.98%	43.98%	41.88%	51.20%	51.20%	50.31%
45 SPM/90%	41.35%	41.35%	39.73%	36.36%	36.36%	31.83%
60 SPM/30%	73.42%	73.42%	73.40%	31.84%	31.84%	31.69%
60 SPM/60%	39.32%	39.32%	39.11%	29.55%	29.55%	26.01%
60 SPM/90%	35.63%	35.63%	37.41%	45.81%	45.81%	36.53%
Average	53.81%	53.81%	53.00%	42.97%	42.97%	40.55%
std	14.55%	14.55%	14.50%	8.48%	8.48%	9.51%

**Table 6:** Performance comparison of the intra-machine fault classification generalization models using different baseline models for OCP-110.

Baseline Model	Partial-Layer Fine-Tuning (PLF)						Full-Layer Fine-Tuning (FLF)					
	Acc1			Acc2			Acc1			Acc2		
	Acc	Recall Rate	F1 Score	Acc	Recall Rate	F1 Score	Acc	Recall Rate	F1 Score	Acc	Recall Rate	F1 Score
I	98.64%	98.63%	98.65%	98.81%	98.78%	98.80%	98.51%	98.49%	98.50%	98.82%	98.80%	98.81%
II	99.53%	99.57%	99.55%	99.76%	99.76%	99.76%	99.87%	99.85%	99.87%	99.90%	99.91%	99.90%
III	99.71%	99.71%	99.70%	99.75%	99.74%	99.74%	99.71%	99.69%	99.70%	99.63%	99.65%	99.74%
IV	99.81%	99.81%	99.81%	99.79%	99.78%	99.78%	99.67%	99.67%	99.66%	99.68%	99.68%	99.67%
V	98.31%	98.28%	98.28%	98.68%	98.67%	98.67%	98.21%	98.17%	98.19%	98.51%	98.49%	98.49%
VI	99.81%	99.81%	99.80%	99.85%	99.87%	99.85%	99.87%	99.88%	99.87%	99.94%	99.93%	99.93%
VII	99.67%	99.67%	99.66%	99.53%	99.50%	99.50%	99.60%	99.62%	99.60%	99.63%	99.60%	99.61%
VIII	100.00%	100.00%	100.00%	98.80%	98.77%	98.77%	100.00%	100.00%	100.00%	99.40%	99.39%	99.39%
IX	98.70%	98.68%	98.67%	97.71%	97.71%	97.71%	98.74%	98.72%	98.71%	97.52%	97.51%	98.13%
X	99.58%	99.55%	99.56%	99.45%	99.41%	99.44%	99.86%	99.86%	99.86%	99.48%	99.48%	99.47%
XI	99.72%	99.70%	99.71%	99.80%	99.80%	99.79%	99.56%	99.57%	99.57%	99.72%	99.73%	99.71%
XII	99.82%	99.81%	99.82%	99.64%	99.63%	99.64%	99.80%	99.81%	99.81%	99.73%	99.74%	99.73%
Average	99.44%	99.44%	99.43%	99.30%	99.29%	99.29%	99.45%	99.44%	99.45%	99.33%	99.33%	99.38%
std	0.53%	0.54%	0.54%	0.63%	0.64%	0.63%	0.58%	0.59%	0.59%	0.68%	0.69%	0.56%

**Table 7:** Performance comparison of the inter-machine fault classification generalization models using different baseline models for G2-110.

Baseline Model	Partial-Layer Fine-Tuning (PLF)						Full-Layer Fine-Tuning (FLF)					
	Acc1			Acc2			Acc1			Acc2		
	Acc	Recall Rate	F1 Score	Acc	Recall Rate	F1 Score	Acc	Recall Rate	F1 Score	Acc	Recall Rate	F1 Score
I	98.73%	98.71%	98.73%	98.32%	98.37%	98.32%	98.27%	98.21%	98.26%	97.81%	97.74%	97.75%
II	99.93%	99.90%	99.92%	99.75%	99.74%	99.76%	99.76%	99.70%	99.72%	99.91%	99.92%	99.92%
III	99.64%	99.66%	99.64%	100.00%	100.00%	100.00%	99.71%	99.68%	99.69%	99.50%	99.51%	99.49%
IV	97.53%	97.60%	97.53%	99.03%	99.00%	99.00%	99.14%	99.15%	99.12%	99.45%	99.47%	99.46%
V	98.31%	98.25%	98.28%	98.19%	98.13%	98.11%	98.68%	98.64%	98.66%	98.64%	98.60%	98.61%
VI	99.92%	99.91%	99.91%	99.83%	99.80%	99.82%	99.91%	99.91%	99.92%	99.70%	99.71%	99.71%
VII	99.93%	99.90%	99.92%	100.00%	100.00%	100.00%	99.93%	99.90%	99.92%	100.00%	100.00%	100.00%
VIII	99.70%	99.72%	99.71%	99.83%	99.83%	99.83%	99.58%	99.58%	99.58%	99.84%	99.86%	99.85%
IX	99.12%	99.15%	99.13%	97.60%	97.56%	97.56%	99.18%	99.18%	99.18%	97.30%	97.20%	97.20%
X	99.68%	99.61%	99.63%	99.59%	99.56%	99.58%	99.92%	99.91%	99.91%	99.66%	99.64%	99.64%
XI	99.82%	99.81%	99.82%	99.86%	99.86%	99.87%	99.60%	99.59%	99.60%	99.14%	99.15%	99.17%
XII	99.13%	99.09%	99.10%	99.48%	99.46%	99.45%	99.28%	99.22%	99.23%	98.72%	98.82%	98.79%
Average	99.29%	99.28%	99.28%	99.29%	99.28%	99.28%	99.41%	99.39%	99.40%	99.14%	99.13%	99.13%
std	0.73%	0.72%	0.73%	0.78%	0.79%	0.80%	0.50%	0.51%	0.51%	0.83%	0.86%	0.85%

**Table 8:** Performance comparison of the inter-machine fault classification generalization models using different baseline models for G2-160.

Baseline Model	Partial-Layer Fine-Tuning (PLF)						Full-Layer Fine-Tuning (FLF)					
	Acc1			Acc2			Acc1			Acc2		
	Acc	Recall Rate	F1 Score	Acc	Recall Rate	F1 Score	Acc	Recall Rate	F1 Score	Acc	Recall Rate	F1 Score
I	98.36%	98.28%	98.23%	98.89%	98.91%	98.95%	97.05%	97.03%	97.03%	98.92%	98.90%	98.89%
II	99.27%	99.19%	99.30%	99.94%	99.94%	99.94%	99.61%	99.58%	99.59%	99.87%	99.85%	99.85%
III	99.70%	99.57%	99.54%	99.62%	99.60%	99.61%	99.69%	99.66%	99.67%	99.77%	99.76%	99.74%
IV	99.19%	99.10%	99.11%	99.70%	99.68%	99.69%	98.61%	98.52%	98.51%	98.74%	98.60%	98.68%
V	98.66%	98.61%	98.62%	98.91%	98.88%	98.79%	98.56%	98.48%	98.45%	98.89%	98.90%	98.83%
VI	99.63%	99.59%	99.62%	99.91%	99.90%	99.91%	99.78%	99.78%	99.77%	99.94%	99.94%	99.94%
VII	99.40%	99.39%	99.39%	99.76%	99.72%	99.73%	99.47%	99.48%	99.48%	99.78%	99.79%	99.78%
VIII	99.38%	99.36%	99.35%	99.56%	99.52%	99.54%	99.37%	99.37%	99.35%	99.72%	99.70%	99.70%
IX	97.73%	97.55%	97.57%	97.77%	97.73%	97.67%	98.35%	98.35%	98.29%	96.99%	97.00%	96.93%
X	99.51%	99.47%	99.48%	99.05%	98.98%	99.01%	99.61%	99.58%	99.59%	99.90%	99.88%	99.88%
XI	99.86%	99.84%	99.85%	99.65%	99.66%	99.64%	99.86%	99.84%	99.85%	99.62%	99.63%	99.60%
XII	98.36%	98.25%	98.26%	98.52%	98.49%	98.43%	98.53%	98.57%	98.57%	98.78%	98.73%	98.73%
Average	99.09%	99.02%	99.03%	99.27%	99.25%	99.24%	99.04%	99.02%	99.01%	99.24%	99.22%	99.21%
std	0.63%	0.66%	0.67%	0.63%	0.64%	0.66%	0.80%	0.80%	0.81%	0.82%	0.82%	0.83%

**Table 9:** Comparison of the accuracies of cross-machine generalization models across four CNN architectures.

Fine-Tuning Method	PLF		FLF	
Sensor	Acc1	Acc2	Acc1	Acc2
Model No.	OCP-110 + G2-110 + G2-160 (Average of 27 accuracies)			
Baseline Model I	98.58% ± 1.09%	98.63% ± 1.30%	97.98% ± 1.40%	98.44% ± 1.53%
Baseline Model II	99.58% ± 0.46%	99.87% ± 0.22%	99.74% ± 0.42%	99.88% ± 0.19%
Baseline Model III	99.63% ± 0.57%	99.80% ± 0.27%	99.67% ± 0.46%	99.63% ± 0.55%
Baseline Model IV	98.88% ± 1.23%	99.51% ± 0.69%	99.20% ± 1.06%	99.30% ± 0.81%
Baseline Model V	98.67% ± 1.16%	98.58% ± 1.26%	98.43% ± 1.16%	98.65% ± 1.26%
Baseline Model VI	99.72% ± 0.39%	99.82% ± 0.26%	99.87% ± 0.21%	99.86% ± 0.29%
Baseline Model VII	99.65% ± 0.45%	99.76% ± 0.38%	99.67% ± 0.44%	99.79% ± 0.28%
Baseline Model VIII	99.64% ± 0.65%	99.71% ± 0.31%	99.64% ± 0.55%	99.70% ± 0.47%
Baseline Model IX	98.79% ± 1.13%	97.30% ± 1.65%	98.83% ± 1.09%	97.21% ± 1.66%
Baseline Model X	99.57% ± 0.67%	99.37% ± 0.57%	99.73% ± 0.50%	99.41% ± 1.11%
Baseline Model XI	99.68% ± 0.49%	99.79% ± 0.28%	99.67% ± 0.45%	99.49% ± 0.66%
Baseline Model XII	99.40% ± 0.83%	99.51% ± 0.51%	99.15% ± 0.96%	99.05% ± 0.88%

**Table 10:** Validation of the optimal generalized model using the ST1-110 machine.

Model No.	Model VI	Model II	Model II
Fine-Tuning Strategy	FLF	PLF	FLF
Sensor	Acc1	Acc2	Acc2
30 SPM/60%	100.00%	100.00%	100.00%
30 SPM/75%	100.00%	97.19%	99.44%
30 SPM/90%	100.00%	100.00%	100.00%
45 SPM/60%	100.00%	99.48%	99.48%
45 SPM/75%	100.00%	100.00%	100.00%
45 SPM/90%	100.00%	99.33%	97.33%
60 SPM/60%	99.37%	100.00%	98.74%
60 SPM/75%	98.47%	100.00%	100.00%
60 SPM/90%	100.00%	100.00%	98.89%
Overall Average	99.76% ± 0.50%	99.56% ± 0.87%	99.32% ± 0.84%
Retraining Time (s)	1889	523	779

## Data Availability

Data is unavailable due to the Non-Disclosure Agreement.
